# Genome-wide identification, phylogeny and expression analysis of *Hsf* gene family in *Verbena bonariensis* under low-temperature stress

**DOI:** 10.1186/s12864-024-10612-8

**Published:** 2024-07-29

**Authors:** Xiuliu Yang, Sisi Wang, Ju Cai, Tao Zhang, Dandan Yuan, Yan Li

**Affiliations:** Key laboratory of Plant Resource Conservation and Germplasm Innovation in Mountainous Region (Ministry of Education), College of Life Sciences, Institute of Agro-bioengineering, Guizhou University, Guiyang, 550025 Guizhou Province China

**Keywords:** *Verbena bonariensis*, *Hsf* gene family, Cold stress, qRT-PCR

## Abstract

**Background:**

The heat shock transcription factor (*Hsf*) is a crucial regulator of plant stress resistance, playing a key role in plant stress response, growth, and development regulation.

**Results:**

In this study, we utilized bioinformatics tools to screen 25 *VbHsf* members, which were named *VbHsf1*-*VbHsf25*. We used bioinformatics methods to analyze the sequence structure, physicochemical properties, conserved motifs, phylogenetic evolution, chromosome localization, promoter *cis*-acting elements, collinearity, and gene expression of *Hsf* heat shock transcription factor family members under low-temperature stress. The results revealed that the majority of the *Hsf* genes contained motif1, motif2, and motif3, signifying that these three motifs were highly conserved in the Hsf protein sequence of *Verbena bonariensis*. Although there were some variations in motif deletion among the members, the domain remained highly conserved. The theoretical isoelectric point ranged from 4.17 to 9.71, with 21 members being unstable proteins and the remainder being stable proteins. Subcellular localization predictions indicated that all members were located in the nucleus. Phylogenetic analysis of the *Hsf* gene family in *V. bonariensis* and *Arabidopsis thaliana* revealed that the *Hsf* gene family of *V. bonariensis* could be categorized into three groups, with group A comprising 17 members and group C having at least two members. Among the 25 *Hsf* members, there were 1–3 exons located on seven chromosome fragments, which were unevenly distributed. Collinearity analysis demonstrated the presence of seven pairs of homologous genes in the *VbHsf* gene family. The Ka/Ks ratios were less than one, indicating that the *VbHsf* gene underwent purification selection pressure. Additionally, nine genes in *V. bonariensis* were found to have collinearity with *A. thaliana*. Promoter analysis revealed that the promoters of all *VbHsf* genes contained various types of *cis*-acting elements related to hormones and stress. Based on RNA-seq data, qRT-PCR analysis of six highly expressed genes was performed, and it was found that *VbHsf5*, *VbHsf14*, *VbHsf17*, *VbHsf18*, *VbHsf20* and *VbHsf21* genes were highly expressed at 12 h of low-temperature treatment, and the expression decreased after 24 h, among which *VbHsf1*4 was up-regulated at 12 h of low-temperature by 70-fold.

**Conclusions:**

Our study may help reveal the important roles of *Hsf* in plant development and show insight for the further molecular breeding of *V. bonariensis*.

**Supplementary Information:**

The online version contains supplementary material available at 10.1186/s12864-024-10612-8.

## Background

Heat shock transcription factor (*Hsf*) is an important regulator of plant stress translation, which regulates gene expression by binding to heat shock response elements (HSE) in the promoter regions of target genes, and then controls the transcription and translation process of heat shock proteins, and responds to various biotic and abiotic stresses [1]. Moreover, *Hsf* also plays an important role in plant growth and development [2, 3]. Studies have demonstrated that the typical *Hsf* in eukaryotes consists of five conserved domains. These domains include the DNA-binding domain (DBD), the oligomerization domain (OD), the C-terminal activation domain (CTAD), the nuclear localization signals (NLS), and the nuclear export signals (NES). Among these domains, the DBD and OD are the most conserved and are responsible for accurately identifying and binding to the target genes’ promoters, respectively. The NLS and NES domains ensure the transport of *Hsf* into the nucleus and maintain the appropriate distribution of genes between the nucleus and cytoplasm. The CTAD contains a transcriptional activation function and directly regulates the expression of relevant genes during heat shock. The transcriptional activation function is mediated by a short activating peptide motif known as the AHA motif within the CTAD [4–7] .

*V. bonariensis* belongs to the Verbenaceae family and the genus Verbena [8]. It is commonly used as a background plant in flower borders and is widely employed in landscape construction. In the karst region of Guizhou, *V. bonariensis* demonstrates adaptability to karst soil and plays a crucial role in many scenic spots, park green spaces, and urban greening projects [9]. Currently, it is cultivated in numerous scenic spots such as Huaxi Gaopo Township (Genting Flower Sea), Wild Jade Sea View Area (Pheasant Ping starry sky flowers), Dushan Laran Town, Tongzi Loushanguan,, Xingren Fangmaping, and more. Especially in recent years, Guizhou Province has made significant efforts in developing the ecological industry and implementing province-wide ecological construction and protection systems. This includes the establishment of urban green spaces (with a green coverage rate of 35% in urban built-up areas), wetland parks, forest parks, mountain parks, etc. With its exceptional qualities as a garden ornamental plant, *V. bonariensis* holds immense potential for widespread application.

Low temperatures are a significant factor that hinders plant growth and distribution [10]. The Guizhou karst area specifically faces the challenge of continuous low temperatures and rainy weather below 4℃ in winter, as well as cold problems in late spring. Unfortunately, *V. bonariensis* is not tolerant to low temperatures and exhibits slow growth below 10℃ [11]. It cannot naturally survive winters below 5℃ [11]. To overcome this issue in production, conventional methods such as greenhouse cultivation and the application of plant hormones are often employed to facilitate overwintering. However, these methods require substantial manpower and material resources, increasing the costs associated with raising, managing, and maintaining *V. bonariensis* seedlings. Consequently, the low temperature resistance of *V. bonariensis* has become a bottleneck in its further promotion and application in Guizhou. Therefore, it is crucial to preserve a sufficient reserve of genes related to cold resistance, specifically isolating and functionally analyzing multi-valent genes and transcription factors with a ‘master switch’ function for plant cold resistance breeding [12].

As a tropical crop, *V. bonariensis* has the characteristics of thermophilic but not cold-tolerant, but the research on *Hsf* gene of *V. bonariensis* has not been reported. In this study, the whole genome identification of the *V. bonariensis Hsf* gene family was carried out, and the sequence characteristics, chromosome localization and genome replication events of the gene family members were analyzed by bioinformatics methods. Combined with the transcriptome data of the leaves of *V. bonariensis* under cold stress, the expression pattern of *VbHsf* gene under cold stress was analyzed, which laid a foundation for further study of the cold response mechanism and biological function of *V. bonariensis Hsf* gene family members, and provided a theoretical basis for further study of the role of this family in the regulation of *V. bonariensis* response to stress.

## Results

### Identification and physicochemical analysis of *Hsf* gene family members in *V. bonariensis*

Through identification and screening, a total of 25 *Hsf* gene family members were identified in *V. bonariensis*. These members were designated as *VbHsf1*-*VbHsf25* based on their distribution on chromosomes (Table S1). The amino acid lengths of the VbHsf proteins ranged from 110 to 474 aa, with molecular weights ranging from 12,410.88 to 52,487.92. The theoretical isoelectric points of these proteins ranged from 4.17 to 9.71. Among the 25 members, 18 were classified as acidic proteins and seven were classified as basic proteins. The analysis of protein hydrophilicity and hydrophobicity revealed that the Hsf protein with the weakest hydrophilicity had a value of -0.968, while the Hsf protein with the strongest hydrophilicity had a value of -0.119; both of these proteins were classified as hydrophilic. The instability coefficient indicated that most of the *Hsf* gene family members were unstable proteins (instability coefficient > 40). However, four members, namely *VbHsf8*, *VbHsf13*, *VbHsf19*, and *VbHsf22*, were found to be stable proteins (instability coefficient < 40), with *VbHsf19* having the lowest instability coefficient of 29.60. The fat coefficient ranged from 52.18 to 78.33. Analysis of transmembrane domains showed that only *VbHsf*19 possessed a single transmembrane domain, while the remaining 24 members did not have any transmembrane domains. The subcellular localization of the *VbHsf* proteins was determined to be in the nucleus. Secondary structure prediction revealed that the *Hsf* family mainly consisted of α-helix and random coil structures, comprising more than 67% of the protein’s amino acid composition. In contrast, the proportion of extended chain structures (β-sheet) and β-turn structures was relatively low (Table S2). These findings suggest that α-helix and random coil structures are prevalent in Hsf proteins, while extended chain and β-turn structures are sparsely distributed throughout the protein.

### Analysis of domain composition and conserved motifs of *Hsf* family proteins in *V. bonariensis*

**Fig. 1:**
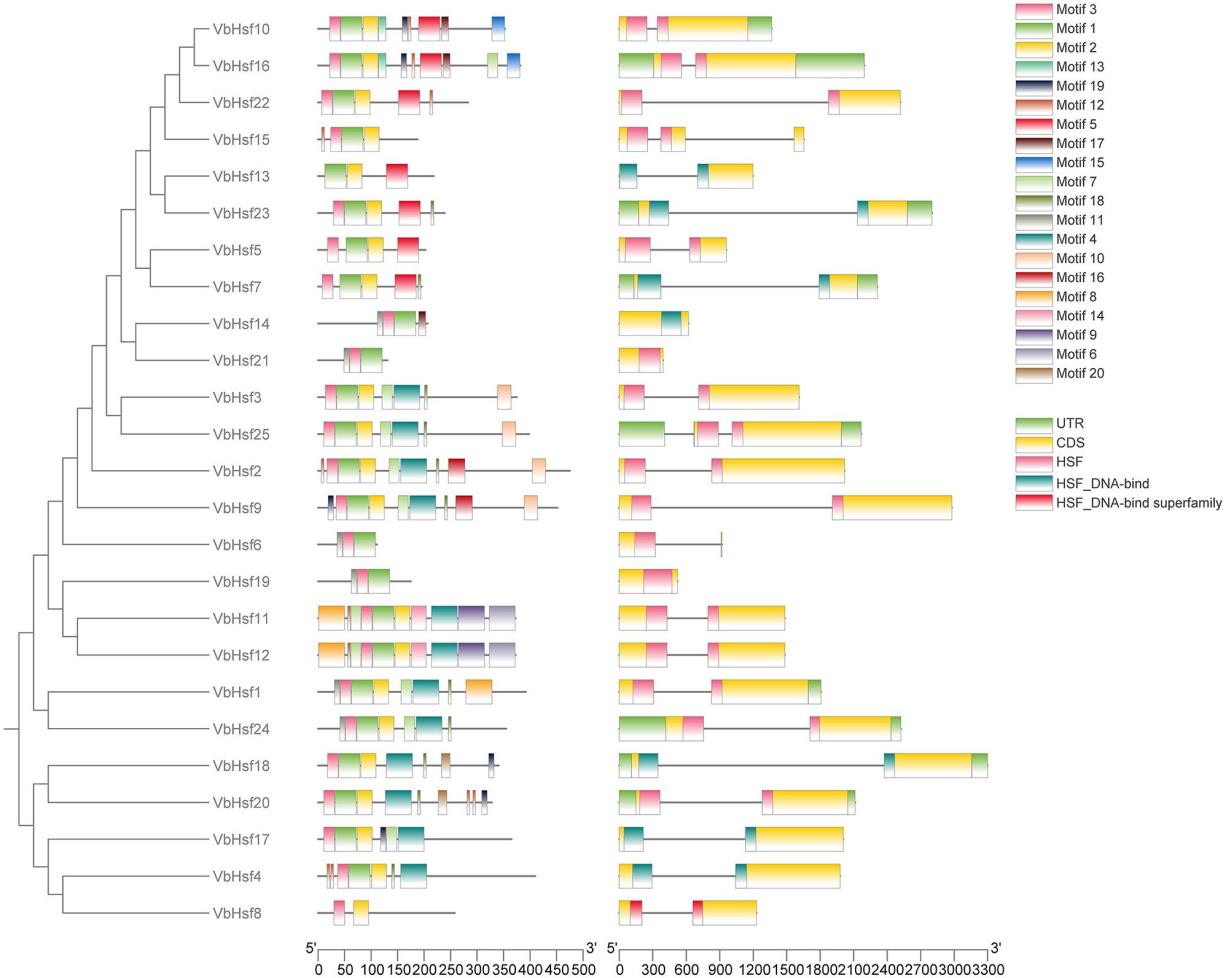
Evolutionary relationship, gene structure and distribution of conserved motifs of *Hsf* gene family members in *V. bonariensis*

To investigate the structural characteristics of *VbHsf* genes, we analyzed the exon, 5’ UTR, and 3’ UTR sequences of 25 *VbHsf* genes. The analysis aimed to explore the genetic structural features of these genes. The findings revealed that *VbHsf* genes generally comprised 1–3 exons. Specifically, *VbHsf6* and *VbHsf13* contained a single exon, *VbHsf15* had three exons, and the remaining *VbHsf* genes had two exons. Among the *VbHsf* genes, only *VbHsf1* and *VbHsf10* had a 3’ UTR sequence, while seven *VbHsf* genes (*VbHsf7*, *VbHsf16*, *VbHsf18*, *VbHsf20*, *VbHsf23*, *VbHsf24*, and *VbHsf25*) possessed both 5’ UTR and 3’ UTR sequences. Notably, the remaining 16 *VbHsf* genes lacked both 5’ UTR and 3’ UTR sequences. Furthermore, closely related genes exhibited similar exon-intron structures (Fig. 1).

**Fig. 2:**
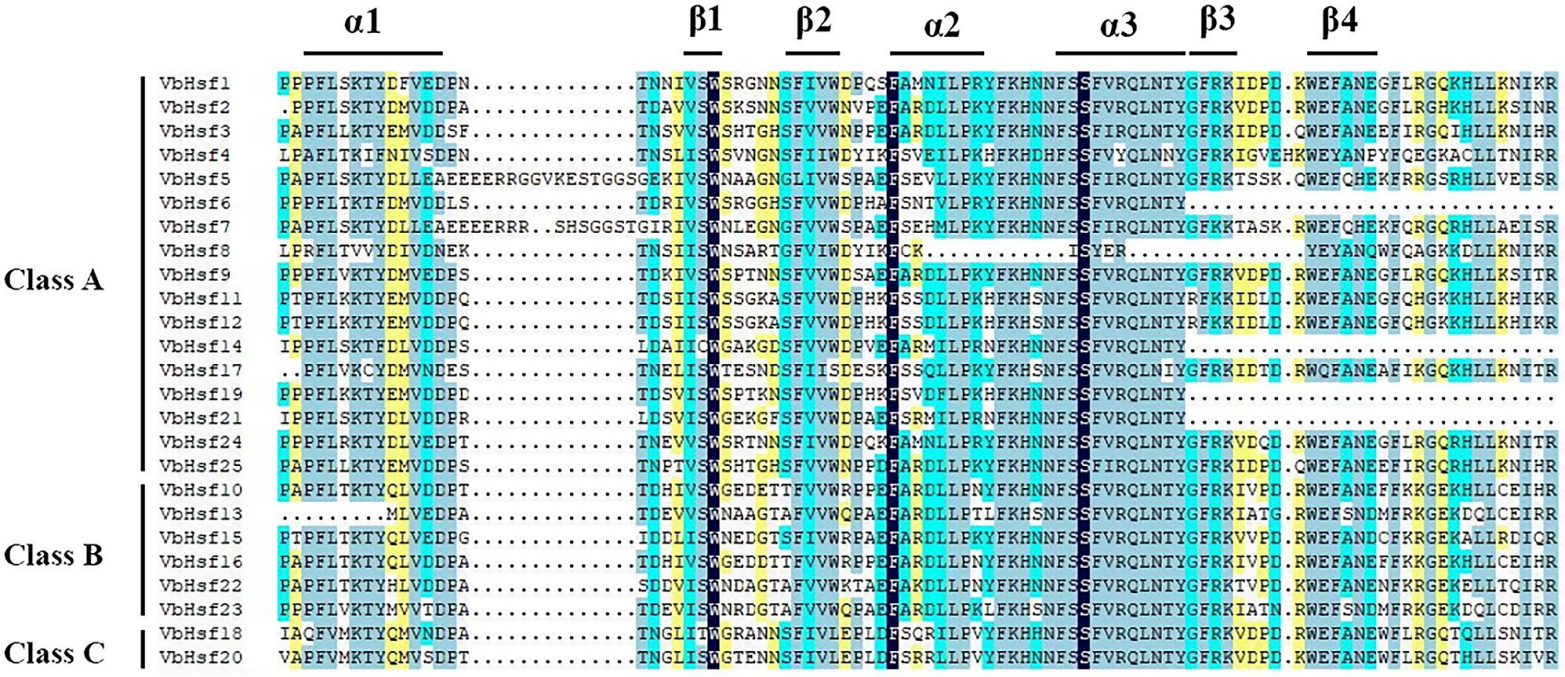
Multiple sequence alignment of DNA binding domains and the HR-A/B regions (OD) of VbHsf

We utilized MEME to conduct a further analysis of conserved motifs in members of the *Hsf* protein family. The findings revealed that the motif types and arrangement in all *VbHsf* genes were largely similar, but some motifs exhibited varying degrees of deletion. Notably, *VbHsf8* contained only two conserved motifs, indicating the most severe deletion. The DNA binding domain (DBD) and oligomerization domain (OD) were identified as the most conserved domains. To investigate the sequence conservation characteristics among members of the *Hsf* gene family, the amino acid sequences of the 25 *Hsf* genes obtained in this study underwent multiple sequence alignment. The alignment demonstrated that all amino acid sequences included a DBD domain composed of motif 3, motif 1, and motif 2. This DBD domain, located at the N-terminus of the *VbHsf* gene, spans approximately 100 amino acids and is characterized by three α-helices (α1-α3) and four β-sheets (β1-β4). These structural features enable specific recognition and accurate localization of heat stress elements. Similar observations have been made in other plant species. Besides DBD, the HR-A/B region, which exhibits a predicted coil-like structure, is crucial for *Hsf*-*Hsf* interaction and trimer formation [13]. Most *VbHsf* genes also possess HR-A/B. Moreover, compared to class B and class C *Hsfs*, class A *Hsfs* have a longer HR-A/B region (Fig. 2, Table S3).

The *Hsf* protein family also includes other domains such as NLS, NES, and AHA. NLS and NES play crucial roles in the intercellular distribution and interaction of *Hsf* genes within the nucleus and cytoplasm [14]. For NLS prediction, we utilized the cNLS Mapper software, while the LocNES software was employed for NES prediction. Most *VbHsfs* were found to contain NES, whereas NLS was absent in class C genes, certain class B genes, and some class A genes. Specifically, 13 (52%) *VbHsfs* contained the NLS domain, while 22 (88%) *VbHsfs* contained the NES domain. The AHA motif is unique to most class A genes. In particular, the AHA motif (motif 10) is exclusive to class A *VbHsf* proteins, and among them, *VbHsf2*, *VbHsf3*, *VbHsf9*, and *VbHsf25* proteins possess the AHA motifs (Table S3).

### Phylogenetic tree and chromosome mapping of *VbHsf* gene family in *V. bonariensis*

**Fig. 3:**
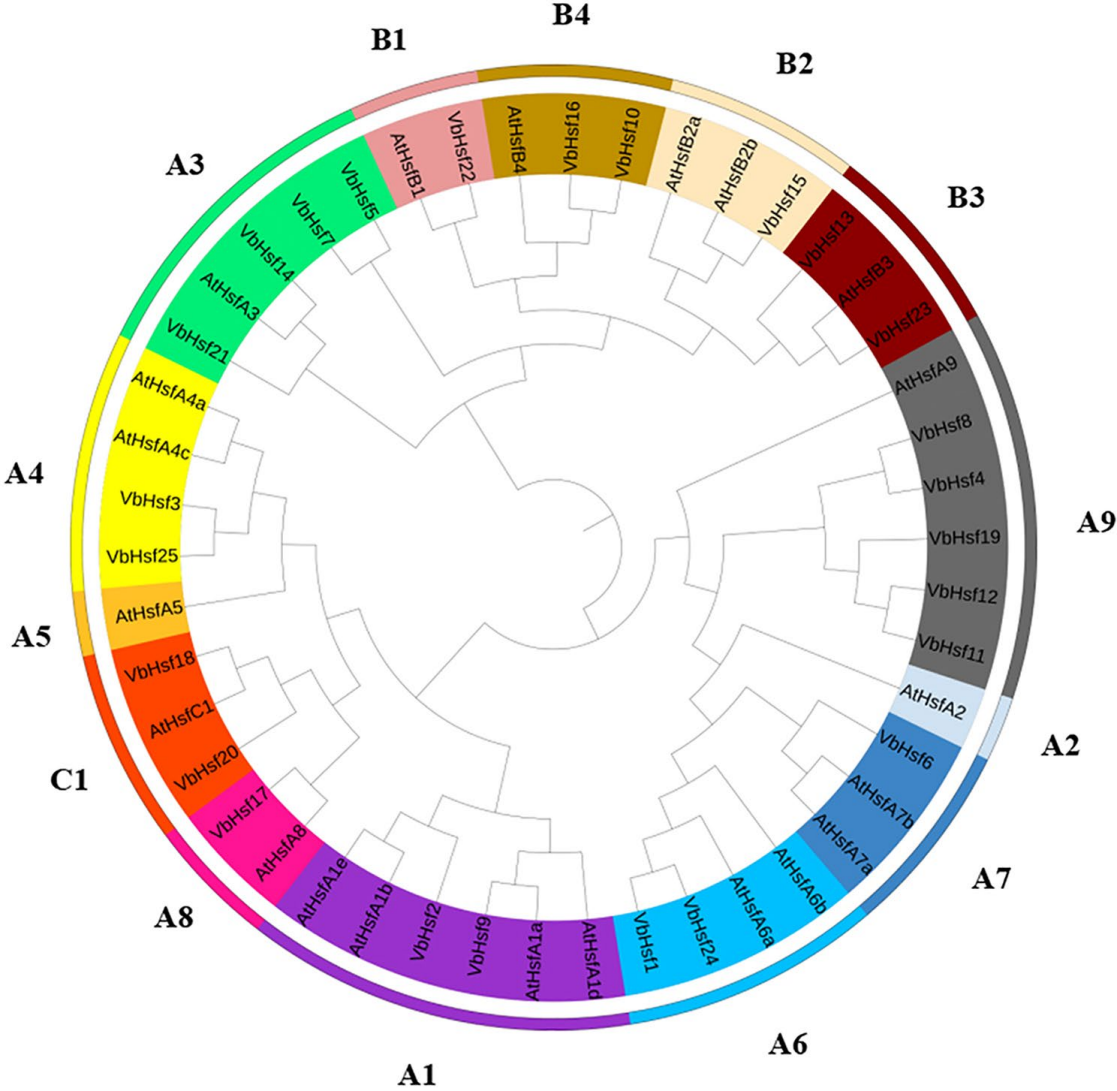
Phylogenetic tree of *Hsf* gene family members between *V. bonariensis* and *A. thaliana*

In order to investigate the homologous evolutionary relationship between *V. bonariensis* and other species, we utilized the *Hsf* gene family of *A. thaliana* as a comparative reference. The identified *Hsf* gene family of *V. bonariensis* (25) and the *Hsf* gene family of *A. thaliana* (21) were then constructed using the maximum likelihood method to create a phylogenetic tree (Fig. 3). The results revealed that the *VbHsf* family genes were categorized into three distinct groups: group A, group B, and group C. Group A consisted of 17 *VbHsf* genes and 15 *AtHsf* genes, group B contained six *VbHsf* genes and five *AtHsf* genes, and the least number of genes was found in group C, which included two *VbHsf* genes and one *AtHsf* gene. Notably, the number of *Hsf* genes in groups A, B, and C were similar between the two species, whereas the number of *Hsf* genes in the three groups differed significantly between *V. bonariensis* and *A. thaliana*.

**Fig. 4:**
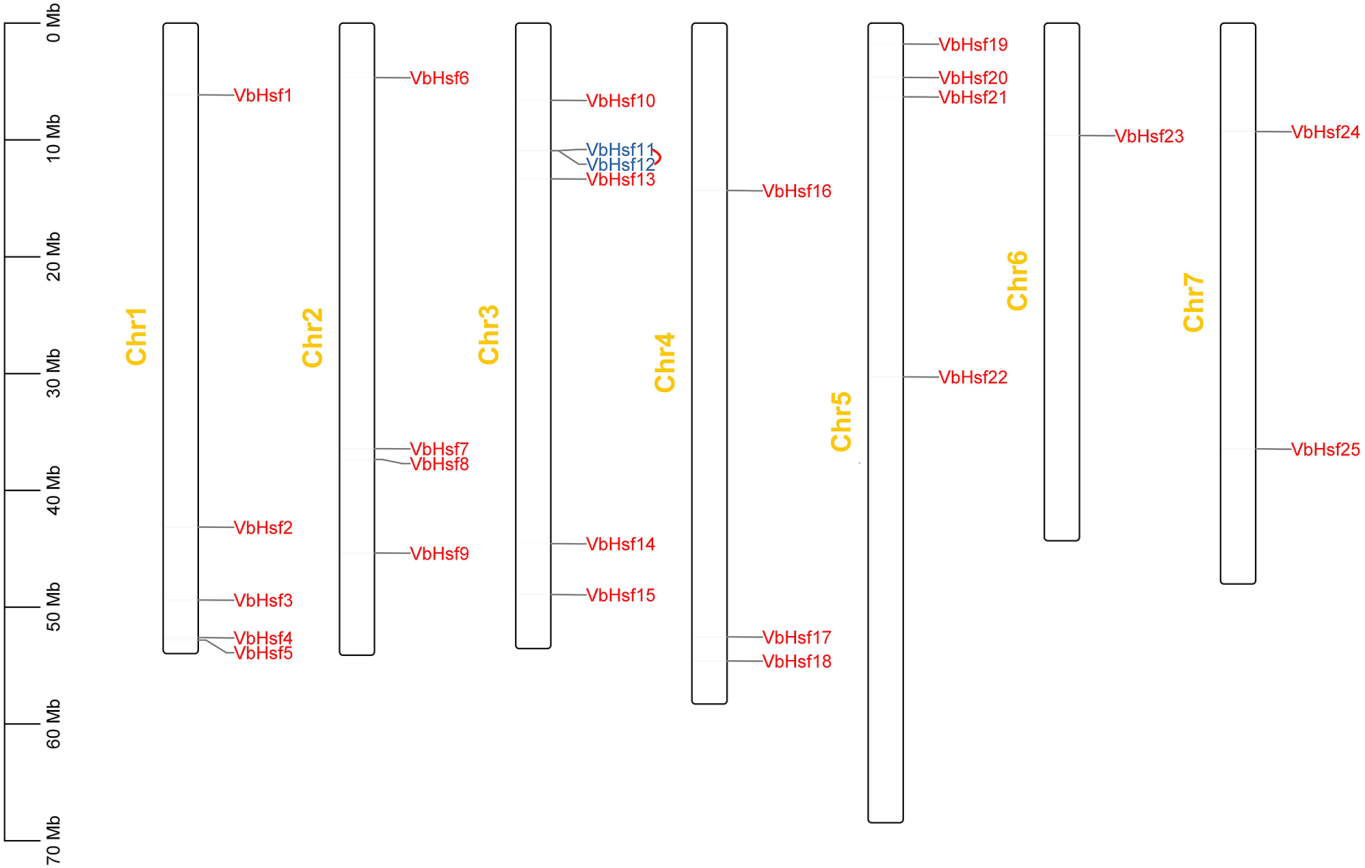
Chromosomal location of *VbHsf* gene family members in *V. bonariensis*

The TBtools tool was employed to create a gene chromosome distribution map based on the chromosome location information of the gene family members. The analysis results (Fig. 4) demonstrated that all *VbHsf* genes were unevenly distributed across 7 chromosome segments. The number of genes on each chromosome ranged from 1 to 6. Chr3 exhibited the highest gene count (6), followed by Chr1 (5). Chr2 and 5 each contained four genes, while Chr4 contained 3 genes. Chr7 contained 2 genes and Chr6 contained 1 gene. Holub defined a chromosomal region with more than two genes within a 200 kb range as tandem repeats [15]. In our study, we identified a pair of tandem repeat genes in *VbHsf*, which are highlighted in blue.

### Analysis of *cis*-acting elements in the promoter of *hsf* gene family in *V. bonariensis*

**Fig. 5:**
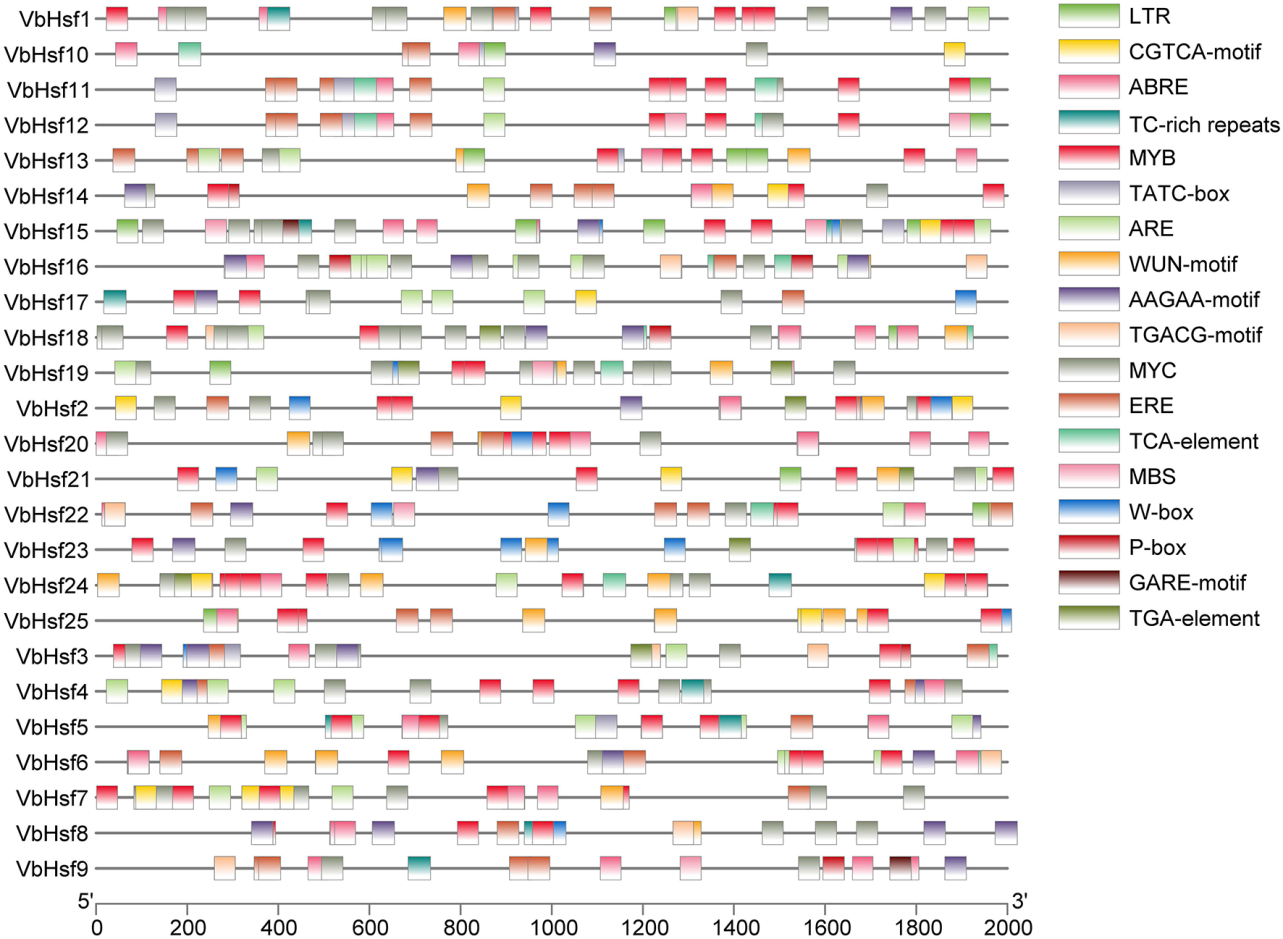
*Cis*-acting element of *V. bonariensis Hsf* gene family

To investigate the potential function of the *VbHsf* gene in *V. bonariensis*, the Plant CARE tool was utilized to predict various *cis*-acting elements within a 2000 bp region upstream of the gene start site. The presence of diverse *cis*-acting elements suggests that different genes may have a range of potential functions. Interestingly, the *VbHsf* family exhibited a variety of *cis*-acting elements in response to environmental and hormone signals (Fig. 5), indicating a complex expression regulation mechanism. For instance, the *cis*-acting element LTR, known to be involved in low temperature response [16], was identified in *VbHsf1*, *VbHsf10*, *VbHsf11*, *VbHsf12*, *VbHsf13*, *VbHsf15*, *VbHsf18*, *VbHsf19*, *VbHsf21*, *VbHsf22*, and *VbHsf25*. Furthermore, eight *VbHsf* genes contained defense and stress response elements (TC-rich repeats). Additionally, 23 *VbHsf* genes possessed drought, high temperature, and low temperature response elements (MYB). The anaerobic sensing element (ARE) was found in 18 *VbHsf* genes, while the WUN-motif and AAGAA-motif (stress response elements) were identified in 17 *VbHsf* genes. Moreover, 24 *VbHsf* genes contained *cis*-acting elements (MYC) related to abscisic acid resistance, drought resistance, freezing resistance, and cold resistance. The cis-acting elements (MBS) associated with drought induction were present in 13 *VbHsf* genes, while the W-box (biotic stress response element) was found in 12 *VbHsf* genes. Jasmonic acid response elements (CGTCA-motif/TGACG-motif) were present in 19 *VbHsf* genes, and abscisic acid signal response elements (ABRE) were identified in 22 *VbHsf* genes. Furthermore, ethylene response elements (ERE) and salicylic acid response elements (TCA-element) were found in 19 and 10 *VbHsf* genes, respectively. The auxin response element (TGA-element) was identified in seven *VbHsf* genes. Additionally, there were nine, six, and foru *VbHsf* genes containing *cis*-acting elements TATC-box, P-box, and GARE-motif, respectively. It is worth noting that the promoters of all *VbHsf* genes contain various types of *cis*-acting elements related to hormones and stress. Among these elements, ABRE and stress response elements MYB and MYC were the most abundant, suggesting that *VbHsfs* may respond to plant hormones and stress, such as salt, drought, low temperature stress, and ABA induction. However, gene expression is regulated by a diverse array of elements, indicating complex regulatory mechanisms that contribute to the diversification of gene functions. Further verification is required to determine the specificity of gene functions.

### Colinearity analysis of *hsf* gene family members in *V. bonariensis*

**Fig. 6:**
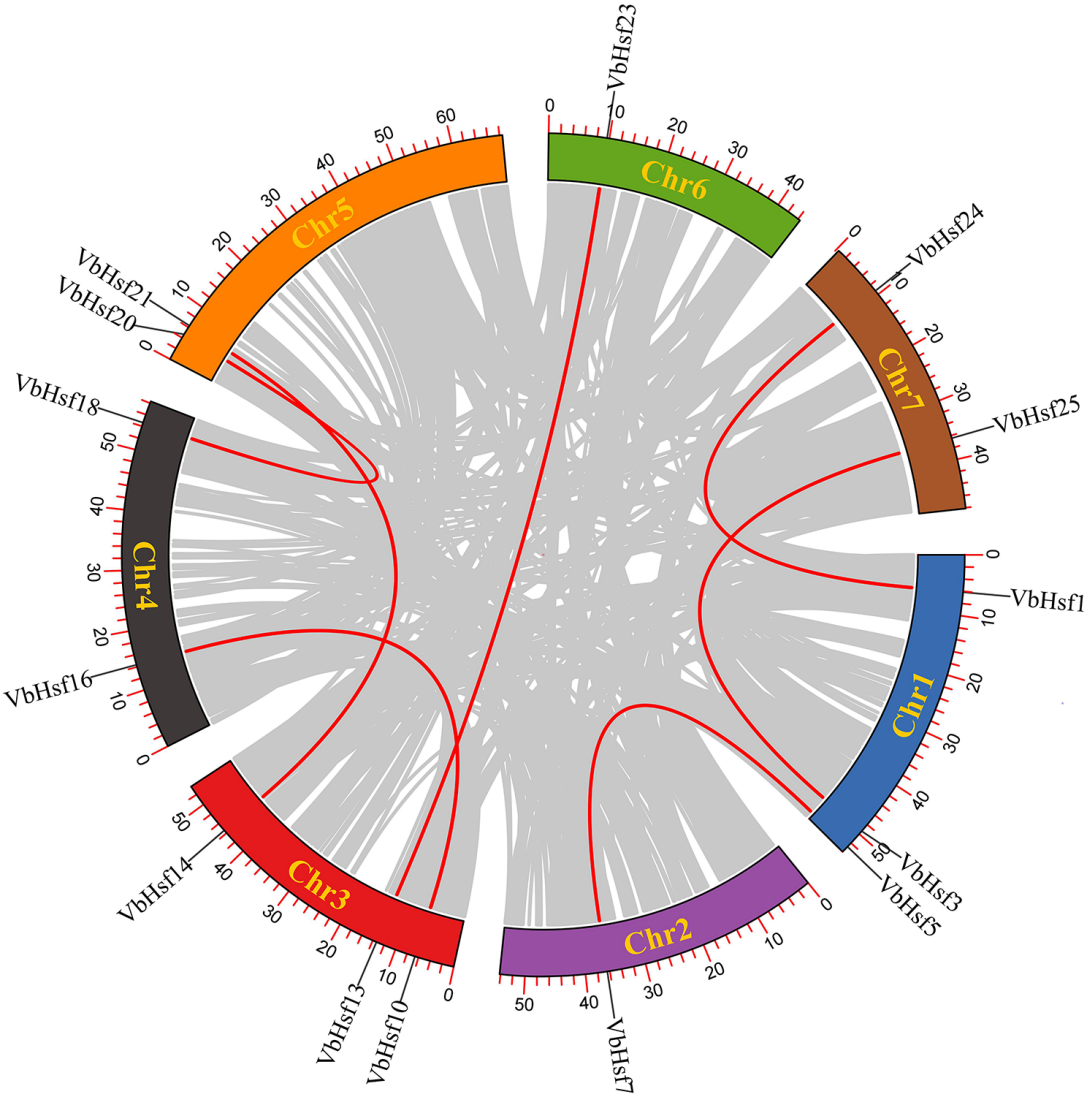
Colinearity relationship of *Hsf* gene family members in *V. bonariensis*

To investigate the conservation of the *Hsf* gene family members in the evolutionary process of *V. bonariensis*, a collinearity analysis was conducted on the *Hsf* gene family members (Fig. 6). The analysis revealed the presence of seven pairs of highly homologous gene pairs within the *Hsf* gene family of *V. bonariensis*. Subsequently, the values of Ka (non-synonymous mutation rate) and Ks (synonymous mutation rate) were calculated for these 7 pairs of highly homologous gene pairs (Table S4). The Ka/Ks ratio is commonly used to determine whether a gene encoding a protein is subject to selective pressure. A Ka/Ks ratio > 1 generally indicates a positive selection effect, while a Ka/Ks ratio = 1 suggests neutral selection, and a Ka/Ks ratio < 1 implies purifying selection. In this study, the Ka/Ks values for all seven pairs of homologous gene pairs in the table were found to be less than one, indicating that these gene pairs evolved under the pressure of purifying selection in *V. bonariensis*.

**Fig. 7:**
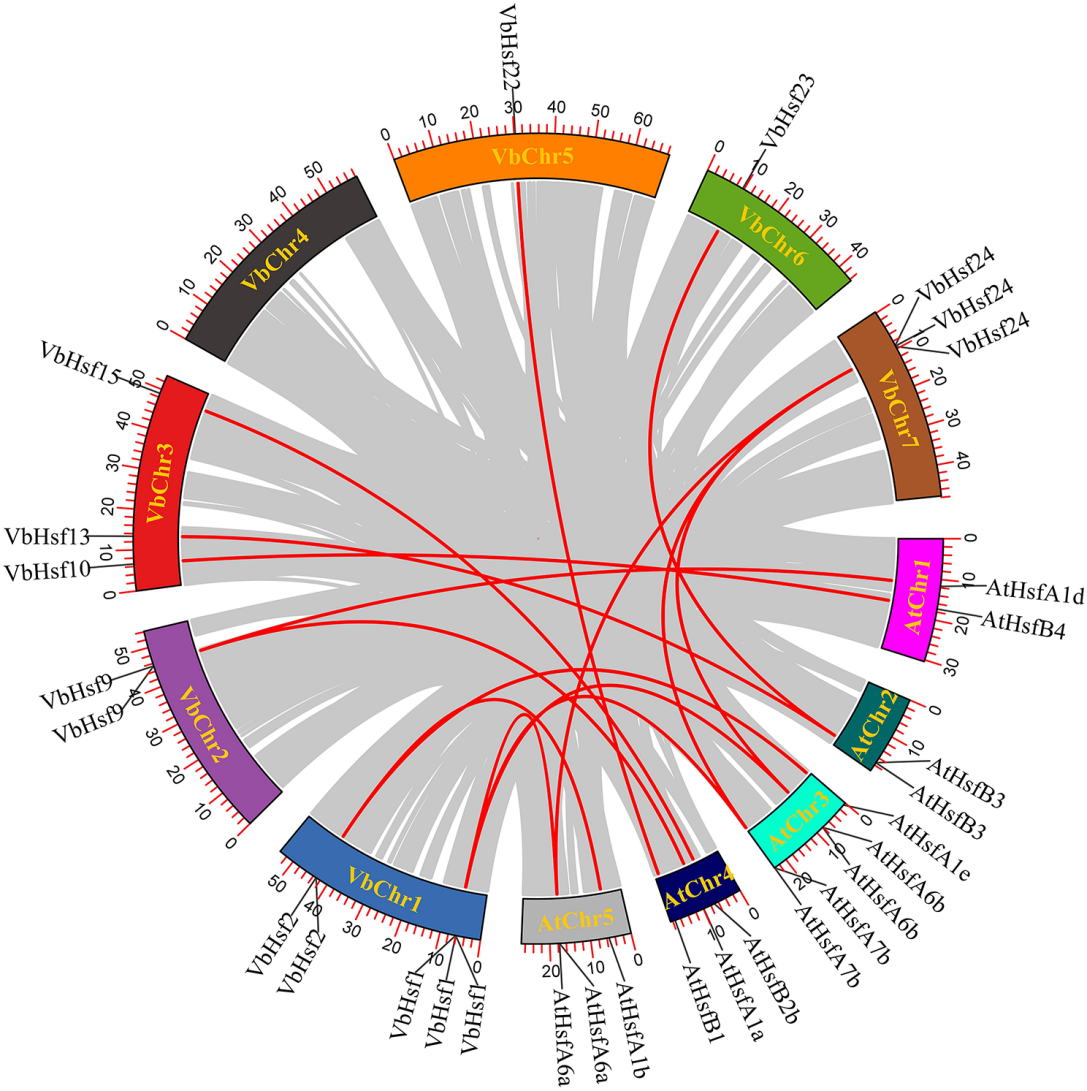
Homology analysis of *Hsf* gene between *V. bonariensis* and *A. thaliana*

To further investigate the origins, evolutionary history, and potential functions of the *Hsf* gene in *V. bonariensis*, we conducted a comparison of the genomic sequences between *V. bonariensis* and *A. thaliana*. We identified a total of 15 pairs of collinear relationships between the two species, with 9 genes from *V. bonariensis* and 11 genes from *A. thaliana* (Fig. 7). Notably, we observed that four genes (*AtHsfA6a*-*VbHsf1/VbHsf24*, *AtHsfA6b*-*VbHsf1/VbHsf24*, *AtHsfA7b*-*VbHsf1/VbHsf24*, and *AtHsfB3*-*VbHsf13/VbHsf23*) in *A. thaliana* exhibited multiple pairings with *V. bonariensis* genes. Similarly, three genes (*VbHsf9*-*AtHsfA1a*/*AtHsfA1d*, *VbHsf2*-*AtHsfA1b*/*AtHsfA1e*, *VbHsf1*-*AtHsfA6a/AtHsfA6b/AtHsfA7b*, and *VbHsf24*-*AtHsfA6a/AtHsfA6b/AtHsfA7b*) in *V. bonariensis* were paired with two or three genes in *A. thaliana*. It is important to note that all these genes belong to the same subfamily, suggesting a shared ancestral origin before subsequent evolution.

### Differences in the expression of *hsf* gene family members in *V. bonariensis*

**Fig. 8:**
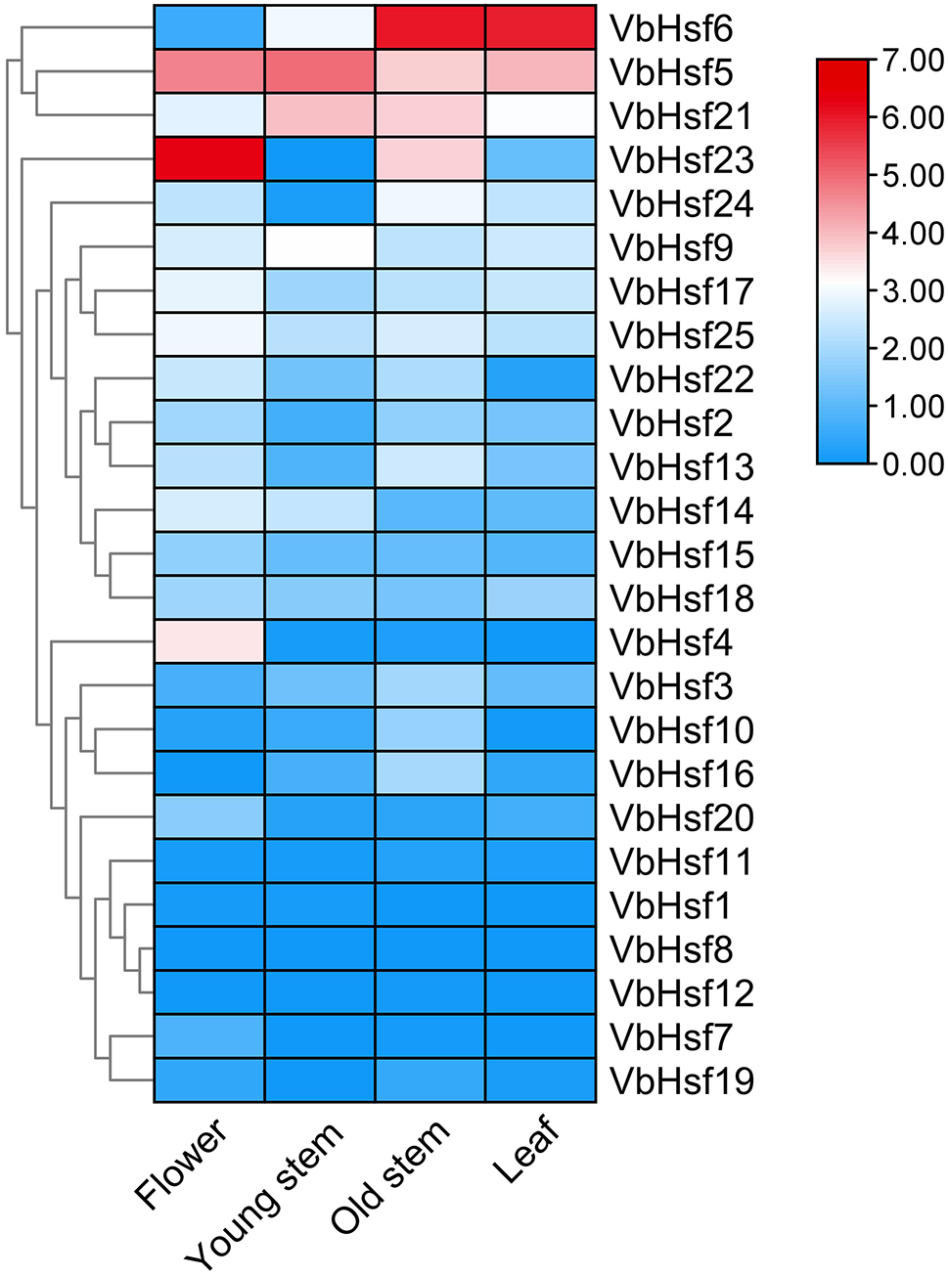
Expression difference of *Hsf* gene family members in different parts of *V. bonariensis*

**Fig. 9:**
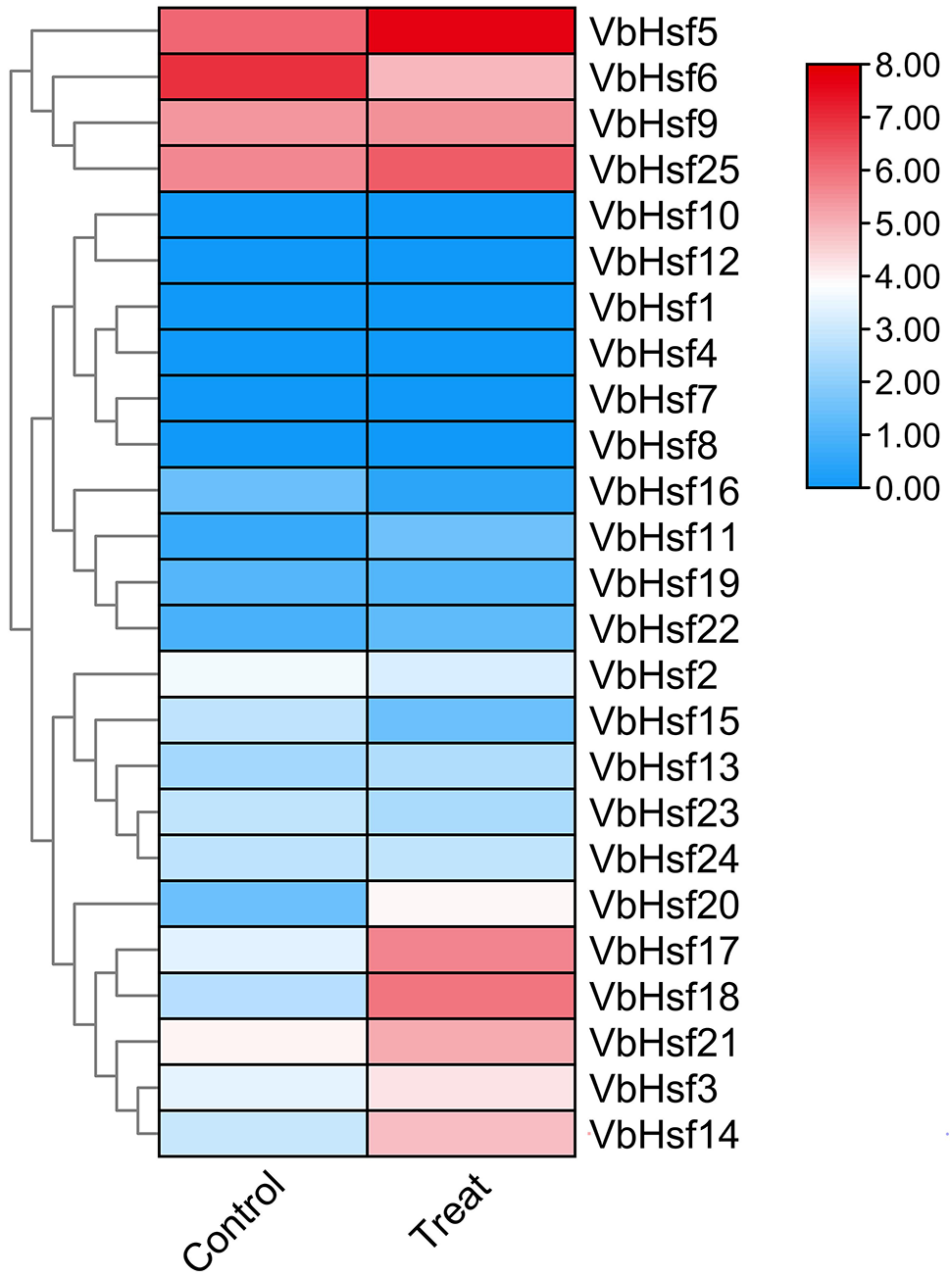
The expression changes of *V. bonariensis* after low temperature treatment

In order to further investigate the regulatory role of *Hsf* gene family members in the growth, development, and response to low temperature stress in *V. bonariensis*, this study analyzed and visualized the transcriptome data of four different parts and low temperature stress conditions from the *V. bonariensis* database (Figs. 8 and 9). The results revealed distinct expression patterns of *VbHsf* in various tissues. Specifically, three *VbHsf* genes (*VbHsf6*, *VbHsf5*, and *VbHsf21*) exhibited high expression levels in almost all tissues, indicating their potential importance in multiple processes. Additionally, all *VbHsf* genes, with the exception of *VbHsf8*, were found to be expressed in at least one of the analyzed tissues. Notably, the significant upregulation of *VbHsf23* in flowers compared to other tissues suggests its potential role in flower development and function.

The expression patterns of the *Hsf* gene family in *V. bonariensis* under low temperature stress can be categorized into three groups. Among these, 13 members showed an increase in expression levels under cold stress, with seven members being significantly up-regulated. On the other hand, six members exhibited a decrease in expression levels, while the remaining 6 members did not respond to cold stress. The up-regulated expression levels of seven *VbHsf* members were found to contain homeopathic elements associated with abiotic stress (LTR, MYB, MYC, WUN-motif), suggesting their potential involvement in the response to cold stress and cold resistance. The distribution of significantly differentially expressed *VbHsf* genes indicated that class A and class C *VbHsf* genes were prominently up-regulated, highlighting their crucial regulatory role in response to low temperature stress. Conversely, the expression of class B *VbHsf* genes was more inhibited under low temperature stress. These findings provide a theoretical foundation for further investigations into the mechanism of low temperature stress response in *V. bonariensis*.

### Examination of the expression of *VbHsf* genes by qPCR under low-temperature stress

**Fig. 10:**
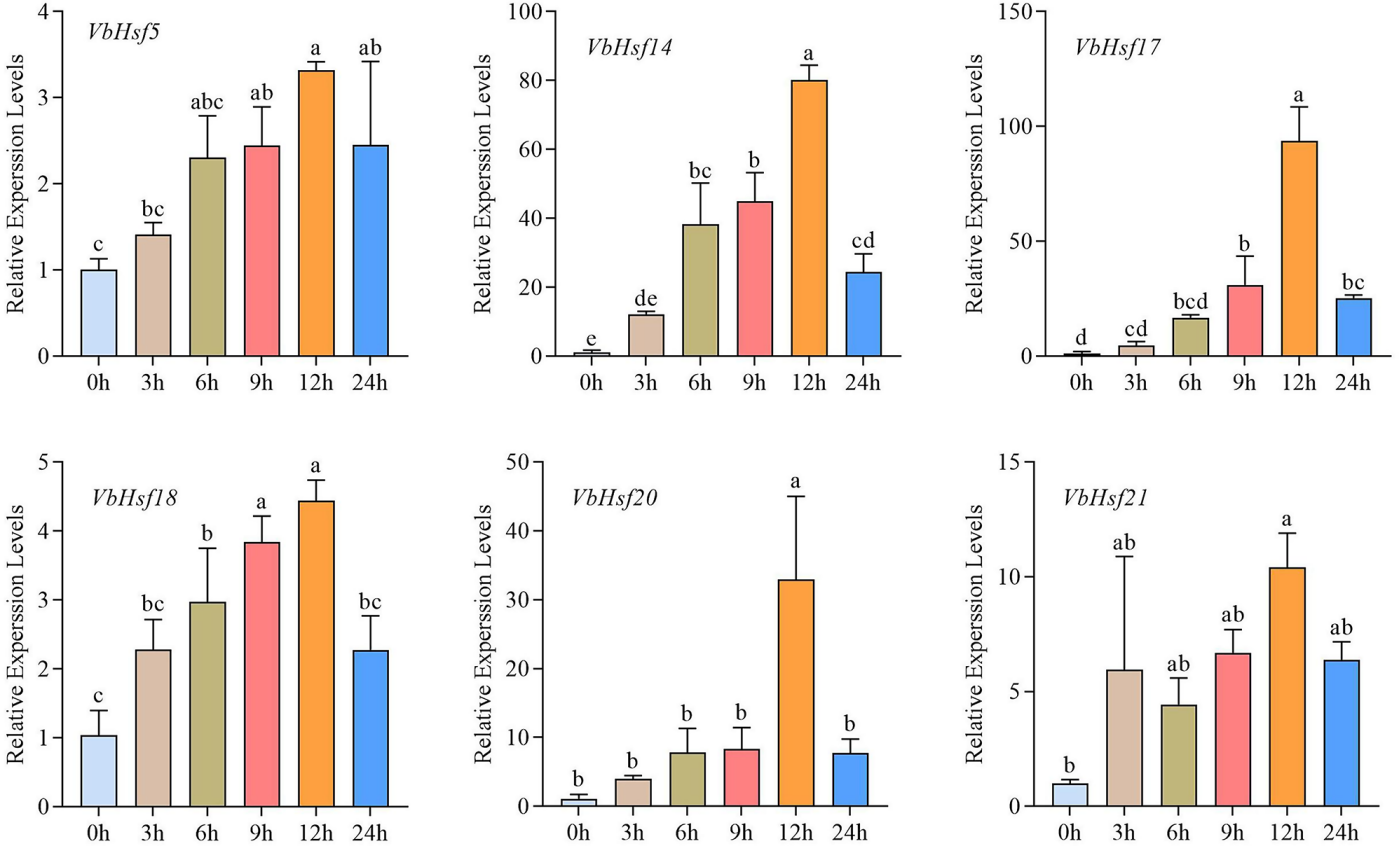
Expression analysis of six *VbHsf* genes in *V. bonariensis* leaves under cold stress. Lowercase letter(s) above the bars indicate significant differences (α = 0.05, LSD) among the treatment

In order to verify the RNA-seq results of *V. bonariensis* under low-temperature stress, we performed qRT-PCR analysis of six highly expressed genes based on RNA-seq data (Fig. 10). It was found that the expression profiles shown by qRT-PCR were similar to those obtained by RNA-seq (Fig. 9). The *VbHsf5*, *VbHsf14*, *VbHsf17*, *VbHsf18*, *VbHsf20* and *VbHsf21* genes showed high expression at 12 h of low temperature treatment, and then the relative expression decreased after 24 h, which showed an overall tendency of increasing and then decreasing; the relative expression of *VbHsf14* and *VbHsf18* was significantly increased at 6 h of treatment; the *VbHsf14* gene had relatively high expression after 3, 6, 9, 12 and 24 h of treatment, which was the highest among all genes. This suggests that these genes may be the key regulators of low- temperature stress response in *V. bonariensis*.

## Discussion

*Hsf* transcription factors are widely distributed in plants and play crucial roles in plant growth, development, and stress response [17]. Extensive studies have been conducted to identify and analyze the members of the *Hsf* family in various plant species. Genome-wide investigations have revealed that *A. thaliana*, *Vitis vinifera* (grapes), *Beta vulgaris* (sugar beets), and *Populus* (poplars harbor) 21, 19, 13, and 28 members of the *Hsf* transcription factor gene family, respectively [18], at the same time, these studies have also shown that the same gene family has some differences in the number of gene family members, gene structure and gene function in different species. However, the *Hsf* gene has not been identified in the whole genome of *V.bonariensis*, and its function has not been reported. Therefore, the launch of the analysis of the *VbHsf* gene family of *V. bonariensis* is conducive to in-depth study of the unique biological functions of the *VbHsf* gene.

The majority of studies have consistently reported that members of the *Hsf* gene family are predominantly hydrophilic proteins and exhibit instability. For example, 17 members of the *Hsf* gene family identified in carnation, were all hydrophilic and mostly unstable proteins. All 25 *Hsf* identified in *V. bonariensis* in this study were also hydrophilic and mostly unstable proteins [19]. The theoretical isoelectric points of the *Hsf* gene family members in most studies were very similar, ranging from 4.65 to 9.36, in the present study, the theoretical isoelectric point of *VbHsf* was found to be 4.17–9.71, which is similar to the results of previous studies [20, 21]. Structural differences among gene family members have been identified as valuable for elucidating functional differentiations across species. For instance, Renjun Qu et al. [22] identified Motifs 1, 2, and 3 in the *Hsf* gene family of *Salvia miltiorrhiza*, excluding *SmHsf*11. Similarly, Lulu Wang et al. [23] discovered that Motifs 1, 2, and 3 were the most widely distributed and conserved in the *Hsf* protein sequence of *Passiflora edulis Sims* (passion fruit). In this study, all *VbHsfs* subpopulations were found to contain motifs 1, 2, and 3 encoding the DBD domain, which displayed a highly conserved triple helix bundle structure. This indicating a high degree of conservation in the *Hsf* gene family during evolution with notable differences. Additionally, this study identified other conserved motifs unique to the members of the *Hsf* gene family in *V. bonariensis*, suggesting the potential importance of these motifs in functional differentiation within this species. The specificity of these structural elements may contribute to the specificity of functions observed in different groups. Furthermore, previous studies have revealed the presence of 2–5 exons in the *Hsf* gene family of *S. miltiorrhiza* [22] and 1–3 exons in the *Hsf* gene family of maize [21], which aligns with the findings of this study regarding the exonic composition of the *Hsf* gene family in *V. bonariensis*.

The phylogenetic tree is a useful tool for elucidating the evolutionary relationships among different species or genes. For instance, Kai Zhao et al. [24] categorized the phylogenetic tree of poplar into group A, group B, and group C based on the grouping in *A. thaliana*. Likewise, Qi Wang et al. [25] divided the *Hsf* gene family in *Arachis hypogaea* (peanut) and *A. thaliana* into three groups (A, B, and C) and 14 subgroups (A9-A1, B4-B1, and C1) in the phylogenetic tree. In this study, the phylogenetic trees of *Hsf* members from *V. bonariensis* and *A. thaliana* were also clustered into three categories and 14 subgroups, mirroring the grouping observed in *A. thaliana Hsf* gene family members [26]. This suggests that homologous genes with similar motif types and arrangement sequences may exhibit functional redundancy, while heterologous genes may share similar functions. Furthermore, the varying number of genes in each group indicates an uneven distribution of *Hsf* gene family members, suggesting a wide range of diversity among them. These genetic differences and diversity likely contribute to the diverse functions of *Hsf* genes, providing valuable resources for studying the *Hsf* gene in *V. bonariensis*.

Replication within gene family members plays a significant role in plant evolution, and collinearity can be used to predict homologous gene sequences during evolutionary processes. In *Cucumis sativus* (cucumber), it was observed that 96% of duplicated gene pairs exhibited Ka/Ks values below 1, indicating strong purification selection [27]. Similarly, in sunflower, the Ka/Ks ratios of *Hsf* gene family members were also lower than one [28]. In this study, it was observed that seven pairs of homologous *Hsf* genes in *V. bonariensis* had Ka/Ks values below one, suggesting that these gene pairs underwent purification selection, which may have contributed to the conservation of the *Hsf* gene structure throughout evolution. Additionally, a collinearity relationship was identified between nine *VbHsfs* and 11 *AtHsfs*, showing some homology, suggesting that they may have evolved from the same ancestor, providing insights into the potential functions of *VbHsf* genes.

Previous studies have emphasized the crucial role of *Hsf* in the response to biotic and abiotic stress, but the identification of cold-related *Hsf* genes has been limited [29]. For instance, Jing Bin et al. [30] demonstrated that certain *OfHsf* members were induced by cold stress in *Osmanthus fragrans*. In *A. thaliana*, Park et al. [31]. identified 30 transcription factors (TFs) that were rapidly induced by low temperatures, referred to as “the first wave of cold-induced TFs,” which included At*Hsf*C1. Furthermore, studies have revealed that the promoters of *Hsf* gene family encompass *cis*-acting elements associated with stress. Qi Zhang et al. [32] found that 20% (six out of 30) of the *Hsf* gene families contained stress-related *cis*-acting elements such as LTR, ARE, and MBS. Pan-Song Li et al. [5] discovered that each soybean *Hsf* member’s promoter carried one or more MYB and MYC *cis*-acting elements. In our investigation, it was observed that the promoters of all *VbHsf* genes contained various stress-related *cis*-acting elements, including LTR, MYB, MYC, WUN-motif, among others. This suggests that *V. bonariensis* potentially regulates plant abiotic stress adaptability. Changwei Shen et al. [33] determined that 44% of *CmHsfs* (16 genes) were significantly up-regulated in pumpkin under cold treatment. Leveraging the genome and transcriptome data of *V. bonariensis*, we analyzed *VbHsf* and found that the expression of *VbHsf* (seven genes) was significantly up-regulated by 28% under low temperature stress.

Plants can respond to abiotic stress and regulate growth and development through tissue-specific gene expression [34, 35]. Liu et al. [36] and Song et al. [37] found that *Hsf* genes were differentially expressed in *Medicago sativa* L., *Chinese cabbage*, respectively. In *V. bonariensis*, we found that *VbHsf* showed specific expression patterns in different tissues, for example, *VbHsf23* was more highly expressed in flowers, suggesting that this gene may play an important role in the growth and development of flowers. *Hsf* has been shown to have an important role in plant response to abiotic stresses [38–40]. Li et al. [41] found in *Dianthus caryophyllus* that most of the *dcahsf* were responsive to heat stress, while some genes were down-regulated under cold stress. In *Cymbidium ensifolium*, the expression of *CeHSF7*, *CeHSF11*, *CeHSF14*, *CeHSF13* and *CeHSF18* were all up-regulated under low-temperature treatment, whereas the expression of *CeHSF15* and *CeHSF21* continued to decrease under low-temperature stress [42]. In *Ginger*, *zohsf* showed an expression pattern that was first up-regulated and then down-regulated under high temperature and high light stress [43]. In this study, six *VbHsf* genes were subjected to qRT-PCR under low-temperature conditions, and the results showed that the expression of six genes, *VbHsf5*, *VbHsf14*, *VbHsf17*, *VbHsf18*, *VbHsf20*, and *VbHsf21*, were upregulated. It was hypothesised that the up-regulated expression of these genes is important for the adaptation of *V. bonariensis* to low-temperature stress and the triggering of cold-response mechanism.

## Conclusion

In this study, a comprehensive and systematic analysis was conducted on the *Hsf* gene family of *V. bonariensis*. A total of 25 members of the *Hsf* gene family were identified. The analysis encompassed gene structure, conserved motifs, conserved domains, chromosome distribution, evolutionary relationship, and distribution of *cis*-acting elements. The findings revealed a highly conserved structure of the *Hsf* gene family, which plays a crucial role in various aspects of the growth and development of *V. bonariensis*. The expression patterns of *VbHsf* genes in various tissues and organs of *V. bonariensis* and their responses to low-temperature stress were investigated. The results showed that the VbHsf gene family plays an important role in the development and response to low-temperature stress in *V. bonariensis*. We demonstrated that six genes, *VbHsf5*, *VbHsf14*, *VbHsf17*, *VbHsf18*, *VbHsf20*, and *VbHsf21*, exhibited positive regulatory roles in the response of *V. bonariensis* to low temperature stress. These findings provide a basis for further exploration of the functions of *Hsf* transcription factors in plant growth, development, and response to low temperature stress, specifically in the context of the *V. bonariensis* species.

## Materials and methods

### Plant material

*V. bonariensis* seeds were purchased from Benary Seed Company, Germany. Seedling conservation, seedling multiplication and hybridisation were carried out in the Seed Resource Nursery of the Key Laboratory of the Ministry of Education of Guizhou University (latitude 26°11′- 26°34′N, longitude 106°27′- 106°52′E). Two month old *V. bonariensis* plants in good growth condition were taken for experimental treatments.

### Identification and physicochemical analysis of *Hsf* gene family members in *V. bonariensis*

First, we obtained the identified 21 *A. thaliana Hsf* protein sequences [44] and the existing genome data of *V. bonariensis* from the *A. thaliana* database (https://www.arabidopsis.org/). Next, we downloaded the hidden Markov model [23]of the *Hsf*-type DBD domain (PF00447) from the Pfam database (https://www.ebi.ac.uk/interpro/entry/pfam). We then conducted preliminary screening and identification using the HMMER software [45]. To ensure the presence of a complete *Hsf* domain, we compared and analyzed the obtained *Hsf* gene family members using tools on the NCBI CDD [46] (https://www.ncbi.nlm.nih.gov/cdd). To predict the length, molecular weight, theoretical isoelectric point, instability coefficient, fat coefficient, and hydrophilic average coefficient of the protein sequence of the *Hsf* gene family members in *V. bonariensis*, we utilized the online tool ProtParam [47] (https://web.expasy.org/protparam/). Additionally, we employed online tools, such as TMHMM2.0 (https://services.healthtech.dtu.dk/services/TMHMM-2.0/), Cell-PLoc (http://www.csbio.sjtu.edu.cn/bioinf/Cell-PLoc-2/), and SOPMA (https://npsa-prabi.ibcp.fr/cgi-bin/npsa_automat.pl?page=npsa_sopma.html), to predict the transmembrane structure characteristics, subcellular localization, and secondary structure of the *Hsf* gene family members in *V. bonariensis*.

### Visualization of gene structure, domain and conserved motif of *hsf* gene family members in *V. bonariensis*

The introns and exons information of the *Hsf* gene family members were obtained from the genome database of *V. bonariensis*. Additionally, the conserved domain information of the *Hsf* gene family members was acquired using the CDD database (https://www.ncbi.nlm.nih.gov/Structure/cdd/wrpsb.cgi) in NCBI. To predict the conserved motif, the online tool MEME (https://meme-suite.org/meme/tools/meme) was utilized, and the visualization of the motif was done through TBtools software. For domain analysis, various software tools were employed to analyze the key conserved domains, namely DBD, OD, NLS, NES, and AHA. To understand the sequence characteristics of the DBD domain and HR-A/B region of *VbHsf*, the amino acid sequence was compared using MUSCLE software, and the sequence was subsequently edited using DNAMAN software [23]. The NLS domain of *VbHsfs* was predicted using the cNLS Mapper software [48] available at https://nls-mapper.iab.keio.ac.jp/cgi-bin/NLS_Mapper_form.cgi. For predicting the NES domain of *VbHsfs*, the LocNES software [49] accessible at http://prodata.swmed.edu/LocNES/LocNES.php was utilized. Lastly, the prediction of the AHA domain was based on the conserved AHA motif sequence FWxxF/L, F/I/L [50].

### Phylogenetic analysis and chromosome mapping of *hsf* gene family members in *V. bonariensis*

The identified *Hsf* family genes of *V. bonariensis* were aligned with the *Hsf* family genes of *A. thaliana* using the multiple sequence alignment tool MUSCLE in MEGA software. The Max Iterations parameter was set to 100, while other parameters were left at their default values [51]. The phylogenetic tree of the *Hsf* family genes in *V. bonariensis* was constructed using the maximum likelihood method ( ML) [52]. The amino acid substitution model used was JTT + G, and the phylogenetic analysis was carried out. To obtain the support rate of each node, the bootstrap method was applied with 1000 repetitions, and the Partial deletion value was set to 80%. The resulting evolutionary tree was edited and enhanced using the online tree diagram tool iTOL [53] (https://itol.embl.de/).The location information of the *Hsf* gene family members was retrieved from the genome database of *V. bonariensis* and visualized using the TBtools software.

### Analysis of *cis*-acting elements of *hsf* gene family members in *V. bonariensis*

The TBtools software was utilized to retrieve the promoter sequence, specifically the 2000 bp region upstream of the initiation codon, from the *V. bonariensis* database. The obtained promoter sequence was then subjected to analysis using the online tool PlantCARE [54] (http://bioinformatics.psb.ugent.be/webtools/plantcare/html/) to predict the *cis*-acting elements that can be recognized and specifically bound by transcription factors. Detailed analysis and numerical statistics were conducted, and the results were visualized using TBtools.

### Colinearity analysis of *hsf* gene family members in *V. bonariensis*

The genome and annotation files of *V. bonariensis* were obtained from the database. The MCScanX software [55] was employed to detect gene duplication events involving the *VbHsf* gene. The mapping of these events was conducted using the TBtools software. To assess the selection pressure on the genes during evolution, the ratio of non-synonymous substitution rate (Ka) and synonymous substitution rate (Ks) was calculated for each non-synonymous site of the identified homologous gene pairs obtained from MCScanX.

### Differences in the expression of *hsf* gene family members in *V. bonariensis*

The expression levels (FPKM) of four different parts (flowers, young stems, old stems, and leaves) and the expression levels of *V. bonariensis* under cold stress were retrieved from the *V. bonariensis* database. Subsequently, the FPKM values were visualized using TBtools.

### qRT-PCR validation

Two-month-old long and healthy *V. bonariensis* plants were used for the cold treatment, and untreated plants with similar growth conditions were used as controls. 4 °C was used as the cold treatment condition, and after 3, 6, 9, 12, and 24 h, the *V. bonariensis* leaves were taken as samples. The samples were placed in sterile, enzyme-free freezing tubes and immediately frozen in liquid nitrogen, and subsequently these samples were stored at -80 °C for RNA extraction. RNA was extracted from the samples by referring to the instructions of the OMEGA Extraction Kit (OMEGA, USA), and cDNA was synthesised by reversing the sample RNAs using the StarScript III RT Kit.The cDNA was used as a template for real-time quantitative PCR assay. qRT-PCR reactions were performed using a qTOWER^3^G system (China) and 2×RealStar Fast SYBR qPCR Mix (GENStar, China). A total volume of 10 µL was prepared for each reaction, including 5 µL of 2×RealStar Fast SYBR qPCR Mix, 0.5 µL each of forward and reverse primers, 1 µL of diluted cDNA, and 3 µL of DEPC water. three replicates were set up for each sample to ensure the reliability of experimental data. Gene-specific primers were designed using the NCBI online website (https://www.ncbi.nlm.nih.gov/tools/primer-blast/). *VbACTIN* was used as an internal reference gene. Data were analysed using the 2^-ΔΔCT method [56].

### Electronic supplementary material

Below is the link to the electronic supplementary material.

Supplementary Material 1

Supplementary Material 2

Supplementary Material 3

Supplementary Material 4

Supplementary Material 5

Supplementary Material 6

Supplementary Material 7

Supplementary Material 8

Supplementary Material 9

Supplementary Material 10

## Data Availability

The datasets used and/or analyzed during the current study are available from the corresponding author on reasonable request.
